# Board of Health

**Published:** 1869-05-15

**Authors:** 


***Board of Health.***

With the beginning of last month (April) the Chicago Board of Health commenced a new era of its existence; the two commissioners appointed at the organization of the Board for the short term of two years, being rotated out of office by the expiration of their term, giving place to two appointed for the full term of six years. The board and the public are to be congratulated upon the re-appointment of one of the old members, Mr. Wm. Giles, to whose intelligence and efficiency we can bear positive testimony. Dr. Schlœtzer, the other appointee, has had the opportunity to add to his professional and scientific acquirements, a thorough familiarity with the practical operations of the Department, having been connected with it since its organization. The Board, as re-organized, has testified its appreciation of the late Sanitary Superintendent, Dr. Jno. H. Rauch, by re-electing him to fill that position, with an increase of compensation commensurate with their appreciation of his value, and now, let us hope the "new brooms will sweep clean," for while much has been done already, the work of sanitary reform is only begun. A little less circumlocution and a little more direct action would greatly enhance the efficiency of the Department.

